# Supplementary material for: Evidence supported by Mendelian randomization: impact on inflammatory factors in knee osteoarthritis
Source: Front Med (Lausanne). 2024 May 28;11:1382836. doi: 10.3389/fmed.2024.1382836 (PMC11165061; doi:10.3389/fmed.2024.1382836)
Supplement: Supplementary file 1 [file Data_Sheet_1.ZIP › data/GCST90274783_5e6/again/783.leaveoneout.pdf]

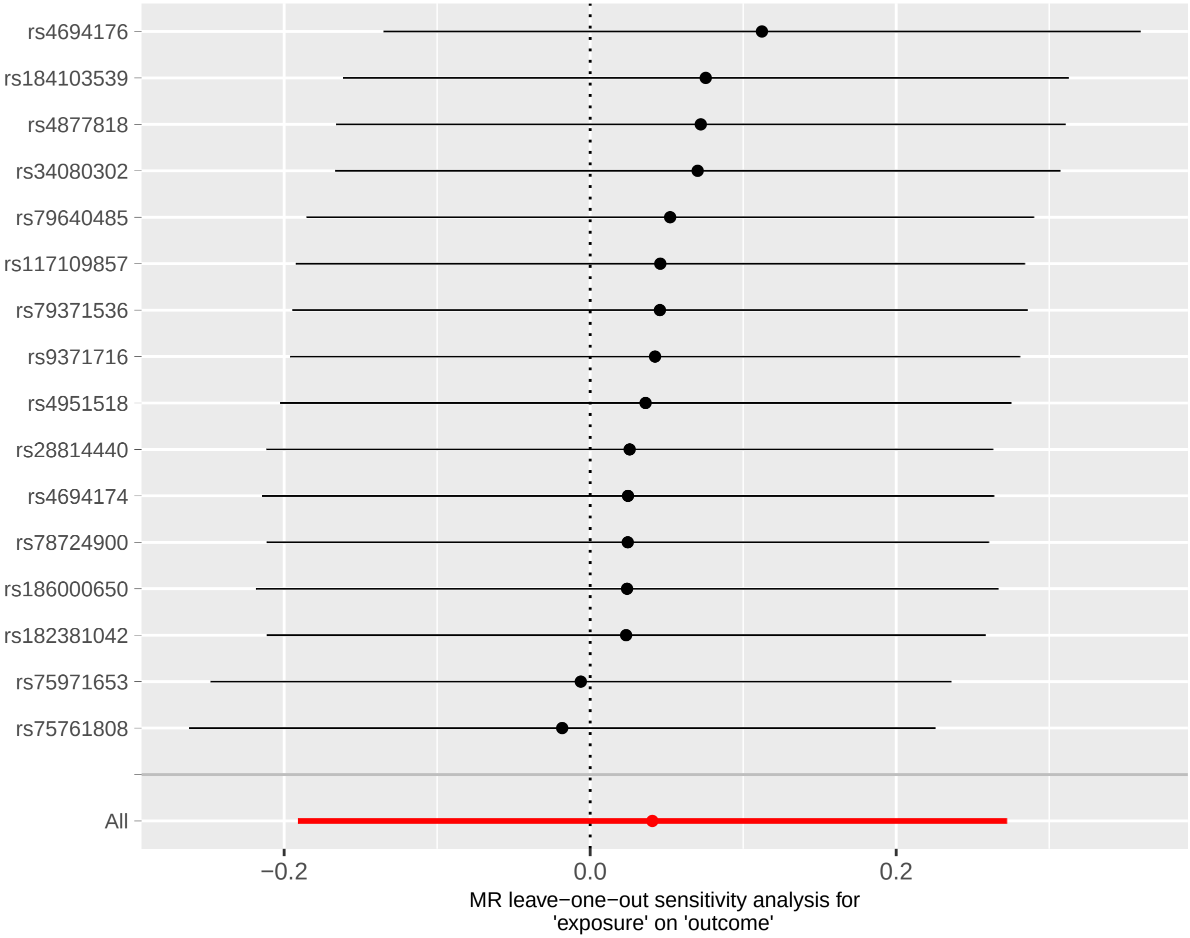
rs4694176
rs184103539
rs4877818
rs34080302
rs79640485
rs117109857
rs79371536
rs9371716
rs4951518
rs28814440
rs4694174
rs78724900
rs186000650
rs182381042
rs75971653
rs75761808
All
−0.2
0.0
0.2
MR leave−one−out sensitivity analysis for
'exposure' on 'outcome'
